# Research on Town Ecological Landscape Planning and Governance Based on Fuzzy Optimization Method of Internet of Things Technology

**DOI:** 10.1155/2022/5159448

**Published:** 2022-05-27

**Authors:** Yu Ying, Kunru Jiang, Meiqi Ren

**Affiliations:** School of Architecture, Yantai University, Yantai, Shandong 264005, China

## Abstract

The current town ecological landscape planning and governance methods are mainly based on the high quality, high energy-saving, and environmental protection effect of the town ecological landscape. How to innovate the town ecological landscape planning and governance process with the help of Internet of things technology and fuzzy optimization method is the current development trend. Based on this, this paper studies the application of Internet of things technology in town ecological landscape planning and management. Firstly, a small town ecological landscape evaluation model based on fuzzy optimization algorithm is proposed. Combined with multivariate matrix transformation function, the authenticity data of ecological landscape are simulated. The original analysis of different types of small town ecological landscape is realized by selecting the multivariate extremum of autocorrelation function curve in the process of planning and governance. Secondly, in the simulation evaluation link, the fuzzy evaluation method is adopted and improved. At the same time, the improved three-dimensional original planning governance model is used to comprehensively analyze the simulation results of three-dimensional landscape planning governance. Finally, by designing fuzzy simulation experiments, the application effects of different Internet of things technologies in town ecological landscape planning and governance are analyzed. The experimental results show that the correlation data indicators of fuzzy optimization methods corresponding to different Internet of things technologies are very different. The application effect of different types of Internet of things technology in ecological landscape planning and governance of small towns is targeted and shows strong regularity.

## 1. Introduction

The research on the planning and governance of small town ecological landscape in China has been carried out very early, and there are many contents involved. It is analyzed from the perspective of the structure of small town ecological landscape, including local landscape simulation, landscape distribution design, and overall planning [1]. In addition, different designers will also participate in the process of town ecological landscape design. From the perspective of town ecological landscape planning and governance, in addition to the three-dimensional layout of the landscape, it will also involve the functional part of the landscape on town greening. In recent years, Internet of things technology has attracted extensive attention. The core content of town ecological landscape planning and governance process is to quantitatively evaluate the effectiveness of green environmental protection, which is of great significance to promote the diversified development of town ecological landscape planning and governance [2]. On the other hand, the domestic green landscape research institute has also carried out the application research of Internet of things technology, and various application modes are emerging, such as the western town ecological landscape model based on analytic hierarchy process [3]. Under this background, this paper studies the application of fuzzy optimization method based on Internet of things technology in town ecological landscape planning and management.

The innovation of this paper is to propose a small town ecological landscape planning and governance model based on improved fuzzy optimization algorithm. Combined with the Internet of things technology, this paper studies the innovative methods of small town ecological landscape in the design process. On this basis, the landscape planning and governance model can not only realize the differential comparison of three-dimensional landscape in different towns, but also make full use of the location differences between the ecological landscapes of each town to analyze the application of different types of Internet of things technology, so as to realize the closed-loop evaluation of the planning and governance quality of the overall ecological landscape of the town. On the other hand, the HQR difference factor is used to quantitatively describe the data matching degree between each comparison column and reference column and the error calculation of standard data, so as to complete the priority ranking of different types of Internet of things technologies in town ecological landscape planning and governance with quantitative indicators, which can efficiently analyze and extract the factors affecting town ecological landscape planning and governance and realize the application value research and analysis of different landscape planning and governance schemes.

Combined with HQR planning and governance model, this study studies the application of Internet of things technology and fuzzy optimization method in town ecological landscape planning and governance, which is mainly divided into four parts. The first part introduces the general framework and research background. The second part introduces the research status of the application of Internet of things technology and town ecological landscape planning and governance. The third part constructs the three-dimensional planning model of small town ecological landscape based on the fuzzy optimization algorithm in the Internet of things technology, adopts the improved fuzzy optimization algorithm in the time domain mode, and constructs the evaluation index system of different fuzzy optimization methods. In the fourth part, the three-dimensional planning model of small town ecological landscape constructed in this paper is simulated and tested, the results are analyzed, and the conclusion is drawn.

## 2. Related Work

At present, there are some problems in the research process of town ecological landscape planning and governance, such as low data useful information rate and insufficient intelligence. Therefore, many experts and scholars have studied the scheme of town ecological landscape planning and governance. According to the location differences of different three-dimensional landscapes in different towns, Kyoung et al. put forward targeted improvement strategies for the planning and governance methods of ecological landscapes in different types of towns [4]. Based on the improvement of the standard unit of the small town ecological landscape planning and governance simulation model, Xiu et al. proposed an adaptive model of small town ecological landscape planning and governance based on neural network algorithm and used the normalization method to collect the nodes of the differences in spatial layout of different small towns. The three-dimensional simulation of small town ecological landscape is realized by neural network algorithm [5]. Huiying et al. found that most towns still follow the traditional ideas of town ecological landscape planning and governance in the process of planning and governance, ignoring the utilization of intelligent information technology and the application of green environmental protection materials [6]. According to information technology and small town architecture, Jiang et al. proposed that attention should be paid to the development and construction of small town ecological landscape planning and governance simulation system based on limiting factors and green environmental protection concept, so as to improve the management and attention to effective data information in the process of small town ecological landscape planning and governance [7]. In order to improve the utilization of existing buildings in the process of town ecological landscape planning and governance, Qingyun et al. proposed an innovative collaborative scheme for town ecological landscape planning and governance based on neural network algorithm and related theories [8]. Enrica et al. found that the current small town ecological landscape planning and management scheme often adopts the characteristics of fixed frame input and put forward the method of cluster analysis and processing of data information based on a specific small town to realize the three-dimensional planning and management of landscape [9]. Josefin et al. put forward a new “end-to-end” small town ecological landscape planning, governance, and transformation system through the research and analysis of the value concept and differences of green environmental protection in different locations of small town ecological landscape planning and governance [10]. Alberto et al. have verified the effectiveness of the system in the process of town ecological landscape planning, governance, and transformation through practice. The results show that the innovative town ecological landscape planning, governance, and transformation system has the advantages of high stability and low cost [11]. Lakshika et al. conducted quantitative comparative analysis of ecological landscape planning schemes in different dimensions according to the intelligent sensing strategy in the Internet of things technology and carried out high-accuracy planning and governance according to its internal data relevance [12].

To sum up, it can be seen that, in the current process of town ecological landscape planning and governance, most of the planning and governance schemes and intelligent matching strategies are obtained by mixed analysis of different types of Internet of things technology and IOT technology [13–15]. On the other hand, although the Internet of things technology has produced a variety of matching application strategies in different types of scenarios, it is difficult to produce greater added value in the specific ecological landscape planning process, which is caused by the direct application without adopting the preferred method [16–19]. Based on this, it is of great practical significance to carry out the research on large-scale management of small town ecological landscape based on fuzzy optimization method based on Internet of things technology.

## 3. Methodology

### 3.1. Basic Application Idea of the HQR Three-Dimensional Planning Model in Town Ecological Landscape Planning and Governance

HQR model is a commonly used three-dimensional planning method, which usually realizes the three-dimensional reconstruction of the landscape through honor in ecological landscape planning [20]. The HQR three-dimensional planning model is mainly divided into two steps in the process of ecological landscape planning and governance of the town, one is the landscape information input link, and the other is the landscape information utilization link [21].

**Figure 1:**
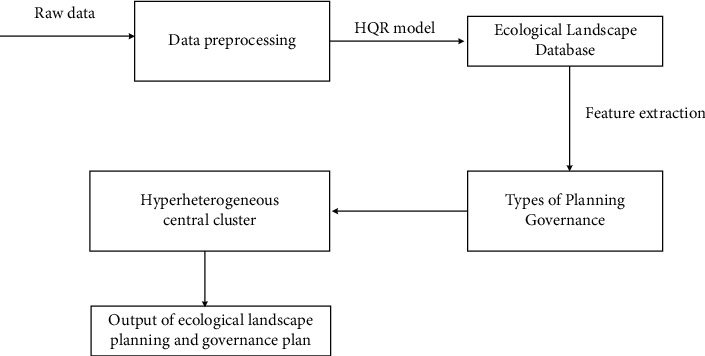
The principle of HQR model in the commonly used ecological landscape planning and governance process.

The process of collecting data from landscape two-dimensional image signals and IOT sensors includes three links. The first process inputs a section of honor or a set of two-dimensional maps of small town landscape. The second link is to infer various parameters in the process of landscape planning through the matching between two-dimensional maps [22]. The principle of HQR model in the process of general ecological landscape planning and governance is shown in Figure 1.

The town ecological landscape planning and governance method based on HQR three-dimensional planning model has been comprehensively developed in recent years, and the three-dimensional space optimization methods in time domain and frequency domain are also emerging. It is difficult for these methods to conduct comprehensive analysis from the perspective of three-dimensional planning [23]. Among many methods for quantitative analysis of ecological landscape simulation results of small towns, HQR three-dimensional planning model has attracted much attention in recent years. This three-dimensional planning method, combined with mathematical methods and information methods, was early applied to solve the problems of three-dimensional restoration of ancient buildings and planning and management of ancient buildings in small towns [24]. Therefore, the planning and management of small town ecological landscape through HQR three-dimensional planning model is to determine the mathematical relationship between many factors of grey system and realize the reconstruction of landscape facilities from 2D to 3D [25, 26].

### 3.2. Fuzzy Optimization Process of Internet of Things Technology in the HQR Three-Dimensional Programming Model

Based on the HQR three-dimensional planning model described in Chapter 3.1, in order to further realize the efficient planning and governance of the town's ecological landscape through the Internet of things technology, this part first selects the HQR three-dimensional planning model based on the time domain method and selects three characteristic parameters related to the coordination of the town's ecological landscape planning and governance. The hierarchical framework and hierarchical subordination of the whole town ecological landscape simulation design system are clearly defined through the research on three aspects: the zero crossing rate of the collected data of the Internet of things sensor of the landscape two-dimensional image signal, the signal waveform conversion, and the calculation of the filtered excitation signal vector. Therefore, in the application of this study to the ecological landscape planning and governance of small towns, the original two-dimensional design information data is transformed into the corresponding matrix for initialization, and then the reference data column is formulated, marked as

(1) $$\begin{array}{c} x_{t}^{\left(0\right)}\left(0\right)=\left\{\frac{x_{1}^{\left(0\right)}\left(0\right),x_{2}^{\left(0\right)}\left(0\right),\ldots ,x_{n}^{\left(0\right)}\left(0\right)}{x_{1}^{\left(0\right)}\left(0\right)-1}\right\}, \end{array}$$

where *i*=1,2,…, *nx*_*t*_^(0)^(0) is different ecological landscape types of small towns.

Secondly, by calculating a sensor data network based on the town landscape planning and governance information and the Internet of things, the absolute difference between the planning factors and governance factors in the town ecological landscape information is calculated, and the formula is expressed as follows:

(2) $$\begin{array}{c} \Delta_{t}\left(i,0\right)=\left|\left\{\frac{x_{t}^{\left(0\right)}\left(i\right)-x_{t}^{\left(l\right)}\left(0\right)}{\sqrt{x_{t}^{\left(0\right)}\left(i\right)+x_{t}^{\left(l\right)}\left(0\right)}}\right\}\right|, \end{array}$$

where *i*=1,2,…, *n*Δ_t_(*i*, 0) is the absolute difference.

**Figure 2:**
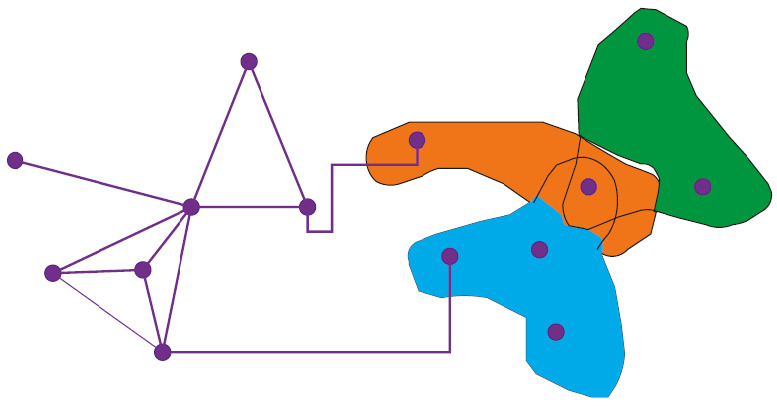
The process of forming the preliminary management plan for the ecological landscape of the small town.

Finally, the correlation degree between the corresponding subfactors and main factors in the HQR three-dimensional planning model is calculated as follows:

(3) $$\begin{array}{c} \zeta_{i}\left(0\right)=\sqrt{\frac{1}{n}\sum_{i=1}^{n}\frac{\underset{t}{\min}\underset{t}{\min}\left|x_{t}^{\left(l\right)}\left(i\right)-x_{t}^{\left(l\right)}\left(0\right)\right|+\zeta \underset{t}{\max}\underset{t}{\max}\left|x_{t}^{\left(l\right)}\left(i\right)-x_{t}^{\left(l\right)}\left(0\right)\right|}{\left|x_{t}^{\left(l\right)}\left(i\right)-x_{t}^{\left(l\right)}\left(0\right)\right|+\zeta \underset{t}{\max}\underset{t}{\max}\left|x_{t}^{\left(l\right)}\left(i\right)-x_{t}^{\left(l\right)}\left(0\right)\right|}}, \end{array}$$

where *∂* represents the three-dimensional planning resolution coefficient of the town's ecological landscape. In this process, the formation process of the preliminary treatment scheme for the town's ecological landscape is shown in Figure 2.

### 3.3. Quantitative Simulation Analysis Process of IOT Technology in the HQR Three-Dimensional Planning Model

In the analytic hierarchy process using the HQR three-dimensional planning model based on IOT (Internet of things) technology, first clarify the problem of selecting the type of ecological landscape planning and governance of the small town, determine the system governance objectives, and then establish the analytic hierarchy process system. Combined with three-dimensional informatics and three-dimensional visualization system, it makes a unified analysis on the change process of relevant signals in the ecological landscape planning and governance of the small town, learns from the existing simulation system of small town ecological landscape planning and governance based on fish network, and applies the feature extraction algorithm to the parameter setting of Internet of things technology.

The specific details of applying the feature extraction algorithm to the technical parameter setting of the Internet of things include the following: The link is divided into data acquisition, data processing, result feedback, and so on. And according to different angles to determine the various indicators of different Internet of things technologies, the system is then divided into different levels. In order to facilitate calculation, it is often explained in the form of block diagram. If there are many factors involved, the hierarchy can be further decomposed. This hierarchy reflects the subordination of various environmental protection building materials, but the importance of each index will not be the same. The current scaling method is mainly the multidimensional marking method, which divides and registers the indicators, assigns values, establishes a matrix, and then extracts useful information from the matrix. By constructing pairwise judgment matrix and matrix mathematical method, the importance of various physical parameters of different Internet of things technologies is ranked. From the perspective of architecture, too many grades will affect the difficulty of judgment. Therefore, in the process of effectively combining the Internet of things technology and HQR three-dimensional planning model, it is necessary to construct the three-dimensional data level of different building materials in this model. Although this structure can reduce the interference of other factors and objectively reflect the difference of influence, a certain degree of nonheterogeneity is bound to occur in the comprehensive comparison. If the results are consistent, the matrix should meet the following requirements:

(4) $$\begin{array}{c} a_{ij}a_{jk}=a_{ik}. \end{array}$$

If any two rows of matrix A are proportional, the scale factor is greater than zero. The consistency test of different environmental protection building materials is expressed by *PP*, and the calculation formula can be expressed as

(5) $$\begin{array}{c} PP=\frac{n}{n-1}\lambda_{\max}. \end{array}$$

After the weight of each index is obtained, the consistency test is carried out, expressed by *b*, and the formula is

(6) $$\begin{array}{c} b_{i}=\sqrt{\sum_{j=1}^{m}b_{ij}a_{j}}. \end{array}$$

**Figure 3:**
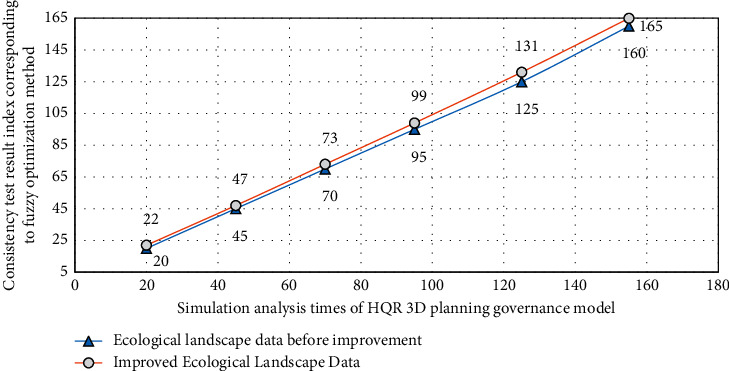
Image of the analysis results of the HQR three-dimensional planning and governance model on the small town ecological landscape data.

The above formula shows that the number of conformance tests needs to be carried out many times before it can be finally determined. Therefore, in the actual calculation, the results of three-dimensional simulation calculation may be inconsistent with the actual situation. The image output after the simulation of the ecological landscape data of the town combined with the improved HQR three-dimensional planning and governance model is shown in Figure 3.

Combined with the method in Figure 2 and the image information in Figure 3, it is not difficult to see that the priority of HQR model for different types of small town ecological landscape planning and governance strategies is different and presents different change laws (the positions of extreme values are inconsistent). This is because the process of small town ecological landscape planning and governance will be affected by subjective factors, which is very prone to objective mistakes. The consistency test cannot be passed, and the physical components of different types of Internet of things technologies are very complex. Therefore, through HQR simulation verification, it is found that this method can promote the interpretation of physical parameters of different types of small town ecological landscape and further improve the simulation quality in the process of small town ecological landscape planning and governance. Therefore, it can not objectively reflect the significance of physical parameters of different Internet of things technologies, and it needs to be evaluated by three-dimensional fuzzy evaluation method. Three-dimensional fuzzy theory has been paid attention to in recent years and has been applied in many fields. This theory is based on set theory. Assuming that the factor domain is represented by *U*, the evaluation level domain is represented by *V*, the membership degree of factor *x* to *v* is represented by *T*, and the fuzzy weight vector is expressed as

(7) $$\begin{array}{c} T=\left(x_{1},x_{2},\ldots ,x_{n}\right), \end{array}$$

where *x* is the factor value of town landscape planning.

### 3.4. Simulation Design of Small Town Ecological Landscape System Based on the HQR Model of Internet of Things Technology

**Figure 4:**
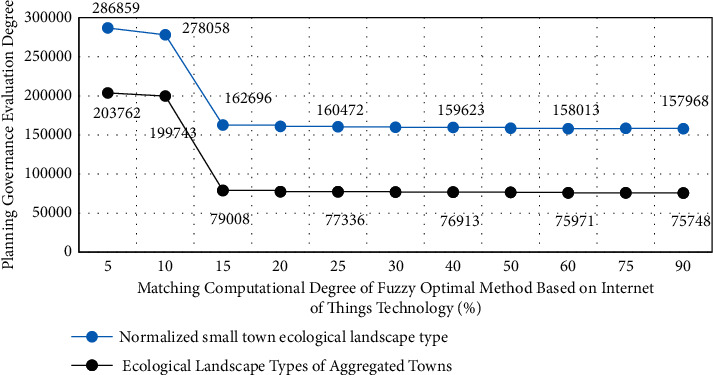
The results of simulation analysis of different types of small town ecological landscape types before optimization.

**Figure 5:**
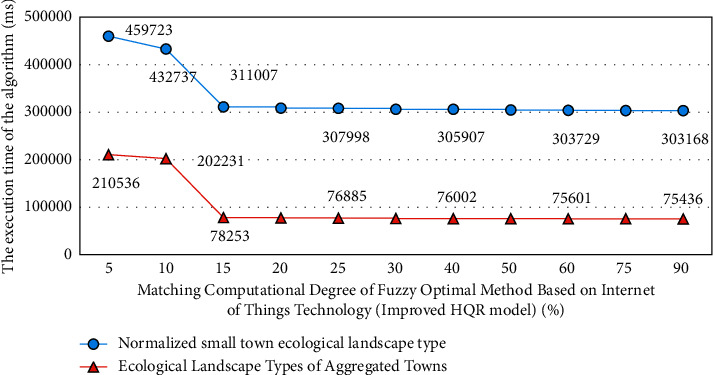
The results of simulation analysis of different types of small town ecological landscape types after optimization.

In the simulation design of the town ecological landscape system, in order to further study the application of different types of Internet of things technologies in the town ecological landscape planning and governance, this paper first summarizes the disadvantages in the process of traditional town ecological landscape planning and governance and combines the characteristics of different types of Internet of things technologies on the basis of HQR three-dimensional model. Then, the physical parameters of ecological landscape in different towns are analyzed through the output and data input of HQR three-dimensional planning and governance model. On the other hand, in the quality evaluation of different types of small town ecological landscape, the grey numbers used are composed of real numbers, and the weights are also different. The simulation analysis results of different types of small town ecological landscape types before optimization are shown in Figure 4, and the optimized results are shown in Figure 5.

As can be seen from Figures 4 and 5, the corresponding change trend before and after the improvement has not changed significantly, but significant changes have taken place in terms of values. This is because, in the small town ecological landscape simulation design system, the original data are transformed into initial values first, and then the reference data column is formulated. Calculate the relationship or correlation degree of different data columns, and then sort the correlation degree.

In the evaluation of the ecological landscape system simulation design of this small town, the physical data of different types of green town ecological landscape have different meanings, so equivalent analysis cannot be carried out. Calculate the absolute difference between each factor and the main factor at the same observation point, and the formula is expressed as

(8) $$\begin{array}{c} \sqrt{\Delta_{t}\left(i,0\right)}=\left\{\frac{x_{t}^{\left(l\right)}\left(i\right)-x_{t}^{\left(l\right)}\left(0\right)}{\sqrt{x_{t}^{\left(l\right)}\left(i\right)+x_{t}^{\left(l\right)}\left(0\right)}}\right\}. \end{array}$$

Finally, the correlation between subfactors and main factors of various types of small town ecological landscape is calculated. In the comprehensive evaluation of simulation results, the ranking problem will be involved in most cases. Each evaluation object needs to be ranked first, so grey comprehensive evaluation is also required.

(9) $$\begin{array}{c} R=\left[\begin{array}{c} \zeta_{1}\left(1\right),\zeta_{1}\left(2\right),\ldots ,\zeta_{1}\left(n\right)\\ \zeta_{2}\left(1\right),\zeta_{2}\left(2\right),\ldots ,\zeta_{2}\left(n\right)\\ \ldots \\ \zeta_{n}\left(1\right),\zeta_{n}\left(2\right),\ldots ,\zeta_{n}\left(n\right) \end{array}\right]. \end{array}$$

Sort by *R* value. The evaluation system is established, and then the HQR three-dimensional programming model proposed in this paper is used to determine the weight of each index, improve the accuracy of index weight, and ensure that the weight distribution is more real. The corresponding HQR three-dimensional planning and governance model in this stage analyzes the differentiated characteristics of the ecological landscape of different towns.

After completing the above steps, it is necessary to establish the evaluation matrix, expressed in *O*, expressed as

(10) $$\begin{array}{c} O={\left(\frac{\sqrt{r_{i,j}}}{\sqrt{r_{i,j-1}}}\right)}_{n\times m}. \end{array}$$

Combined with the previous design, the comprehensive town ecological landscape simulation design evaluation system is established, and then the comprehensive evaluation is carried out. In the application of fuzzy optimization algorithm based on Internet of things technology, a hierarchical fuzzy evaluation vector *C*_1_^*s*^ is calculated, and its formula is

(11) $$\begin{array}{c} C_{1}^{s}=\sqrt{W_{C_{1}}R_{C_{1}}^{s}}, \end{array}$$

where *s*=1,2,…, *p*, and then calculate the secondary evaluation matrix:

(12) $$\begin{array}{c} C_{A}^{s}=\left[\frac{B_{1}^{s},B_{2}^{s},B_{3}^{s},B_{4}^{s}}{\sqrt{B_{1}^{s}+B_{2}^{s}}}\right]. \end{array}$$

In this, *s*=1,2,…, *p*

Then calculate the absolute difference between the subfactors and the main factors of the ecological landscape of different types of green towns at the same time to obtain its correlation error. Calculate the grey correlation coefficient according to the correlation degree between various factors. The calculation formula is

(13) $$\begin{array}{c} \zeta_{s}=\frac{1}{5}\overset{s}{\underset{j=1}{\sum }}B_{sj},\\ P=\left(P_{1},\ldots ,P_{m}\right).\\ Z_{i}=\left(Z_{i1},\ldots ,Z_{im}\right), \end{array}$$

*P* and *Z* are different types of town ecological landscape planning and management schemes.

## 4. Result Analysis and Discussion

### 4.1. Experimental Design Process of Environmental Protection Building Materials in Town Ecological Landscape Planning and Management

**Figure 6:**
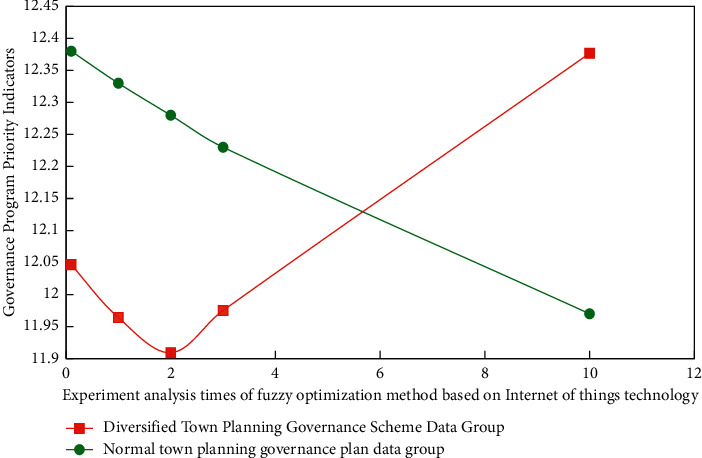
Preliminary experimental results analysis images.

When studying the application of Internet of things technology in town ecological landscape planning and governance, relevant differentiation experiments are designed according to different Internet of things technical parameters and planning and governance strategies. Before the experiment, it is assumed that the primary index weight coefficient has been determined, which is expressed by *a*_1_ and greater than 0. Each secondary indicator will also have its own weight coefficient, represented by *a*_*j*_, and the weight vector is expressed as *A*_1_, which is also greater than zero. The data group of diversified urban planning government plan is set as the experimental group, and the data group of normal urban planning governance plan is set as the control group. In this process, the corresponding experimental results of the fuzzy optimization method based on Internet of things technology for two different town ecological landscape planning and governance schemes are shown in Figure 6.

### 4.2. Experimental Results and Analysis

**Figure 7:**
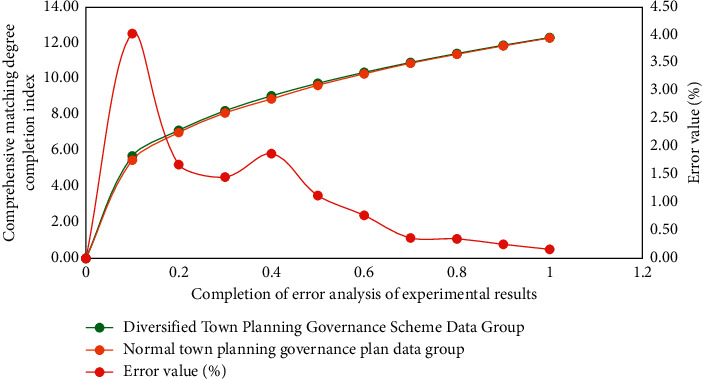
Error analysis of experimental results of simulation model for small town ecological landscape planning and governance.

Figure 7 is the error analysis process of the experimental results of different types of Internet of things technology in the small town ecological landscape planning and governance simulation model. The relevant data of different types of small town ecological landscape experiments are processed by MATLAB software. Because these index data are very large, in order to facilitate calculation, in the analysis of this study, two different town ecological landscape planning and management schemes are processed in blocks.

From the results of Figures 6 and 7, we can know the comprehensive application of fuzzy optimization methods corresponding to different types of Internet of things technologies in the planning and management of ecological landscape of small towns. From the experimental results, it can be seen that similar methods are also used to calculate the evaluation vectors of other indicators, and the difference of grey evaluation matrix is very obvious, and the error can be controlled within 5%. This is because the difference of correlation degree data indicators corresponding to fuzzy optimization methods corresponding to different Internet of things technologies is very obvious. Therefore, the absolute values in the process of ecological landscape planning and governance of different types of small towns are also different. Therefore, the experimental results show that the application effect of different types of Internet of things technologies in ecological landscape planning and governance of small towns is targeted and showed a strong regularity.

## 5. Conclusion

The current town ecological landscape planning and governance model has the problems of large proportion of subjective factors and low intelligence. Based on this, this paper studies the application of fuzzy optimization method based on Internet of things technology in town ecological landscape planning and management. Firstly, a small town ecological landscape evaluation model based on fuzzy optimization algorithm is proposed. Combined with multivariate matrix transformation function, the authenticity data of ecological landscape are simulated. The original analysis of different types of small town ecological landscape is realized by selecting the multivariate extremum of autocorrelation function curve in the process of planning and governance. Secondly, the fuzzy evaluation method is adopted and improved in the simulation evaluation link. At the same time, the improved three-dimensional original planning and governance model is used to comprehensively analyze the simulation results of three-dimensional landscape planning and governance, and the landscape planning and governance model can not only realize the differential comparison of three-dimensional landscapes of different towns in the design, but also make full use of the location differences between the ecological landscapes of each town to analyze the application of different types of Internet of things technology, so as to realize the closed-loop evaluation of the planning and governance quality of the overall ecological landscape of the town. Finally, by designing fuzzy simulation experiments, the application effects of different Internet of things technologies in town ecological landscape planning and governance are analyzed. However, this study only considers the application of Internet of things technology in town ecological landscape planning and governance and does not consider the application of other materials, so more in-depth research can be carried out.

## Data Availability

The data used to support the findings of this study are available from the corresponding author upon request.

## References

[1] Gong J., Cao E., Xie Y., Xu C., Li H., Yan L. (2021). Integrating ecosystem services and landscape ecological risk into adaptive management: insights from a western mountain-basin area, China. *Journal of Environmental Management*.

[2] Armansin N. C., Stow A. J., Cantor M. (2020). Social barriers in ecological landscapes: the social resistance hypothesis. *Trends in Ecology & Evolution*.

[3] Xu J., Zhao H., Yin P., Wu L., Li G. (2019). Landscape ecological quality assessment and its dynamic change in coal mining area: a case study of Peixian. *Environmental Earth Sciences*.

[4] Rai A. C. (2021). Energy performance of phase change materials integrated into brick masonry walls for cooling load management in residential buildings. *Building and Environment*.

[5] Rashad A. M., Khalil M. H., El-Nashar M. H. (2021). Insulation efficiency of alkali-activated lightweight mortars containing different ratios of binder/expanded perlite fine aggregate. *Innovative Infrastructure Solutions*.

[6] Coelho-Júnior H., Silva B. G., Labre C., Loreto P. R., Sommer L. R. (2021). Room-temperature synthesis of earth-abundant semiconductor ZnSiN2 on amorphous carbon. *Scientific Reports*.

[7] Jiang L., Xiong Y., Wang Y., Luo T. (2018). Soundscape Effects on Visiting Experience in City Park: a Case Study in Fuzhou, China. *Urban Forestry & Urban Greening*.

[8] Du Q., Wu C., Ye X., Ren F., Lin Y (2018). Evaluating the effects of landscape on housing prices in urban China. *Tijdschrift voor Economische en Sociale Geografie*.

[9] Dall’Ara E., Maino E., Gatta G., Torreggiani D., Tassinari P. (2018). Green Mobility Infrastructures. A landscape approach for roundabouts’ gardens applied to an Italian case study. *Urban Forestry & Urban Greening*.

[10] Persson J., Wang T., Hagberg J. (2018). Organophosphate flame retardants and plasticizers in indoor dust, air and window wipes in newly built low-energy preschools. *The Science of the Total Environment*.

[11] Gasparotto A., Carraro G., Maccato C. (2018). WO3-decorated ZnO nanostructures for light-activated applications. *CrystEngComm*.

[12] Weerasundara L., Magana-Arachchi D. N., Ziyath A. M., Goonetilleke A., Vithanage M. (2018). Health risk assessment of heavy metals in atmospheric deposition in a congested city environment in a developing country: kandy City, Sri Lanka. *Journal of Environmental Management*.

[13] Hampe A., Alfaro-Sánchez R., Martín-Forés I. (2020). Establishment of second-growth forests in human landscapes: ecological mechanisms and genetic consequences. *Annals of Forest Science*.

[14] Wu Y., Huang L., Zhao C., Chen M., Ouyang W. (2021). Integrating hydrological, landscape ecological, and economic assessment during hydropower exploitation in the upper Yangtze River. *The Science of the Total Environment*.

[15] Jiang P., Li M., Lv J. (2019). The causes of farmland landscape structural changes in different geographical environments. *The Science of the Total Environment*.

[16] Dai X., Zhuang D. (2019). Geographic planning and design of marine island ecological landscape based on genetic algorithm. *Journal of Coastal Research*.

[17] Jahanishakib F., SalmanmahinY A., Mirkarimi S. H., Poodat F. (2021). Hydrological connectivity assessment of landscape ecological network to mitigate development impacts. *Journal of Environmental Management*.

[18] Zegers G., Arellano E., Östlund L. (2020). Using forest historical information to target landscape ecological restoration in Southwestern Patagonia. *Ambio*.

[19] Camacho L. F., Barragán G., Espinosa S. (2021). Local ecological knowledge reveals combined landscape effects of light pollution, habitat loss, and fragmentation on insect populations. *Biological Conservation*.

[20] Rosas Y. M., Peri P. L., Lencinas M. V., Martinez Pastur G. (2019). Potential biodiversity map of understory plants for Nothofagus forests in Southern Patagonia: analyses of landscape, ecological niche and conservation values. *The Science of the Total Environment*.

[21] Jafari S. M., Nikoo M. R. (2019). Developing a fuzzy optimization model for groundwater risk assessment based on improved DRASTIC method. *Environmental Earth Sciences*.

[22] Han S. I. (2019). Fuzzy supertwisting dynamic surface control for MIMO strict-feedback nonlinear dynamic systems with supertwisting nonlinear disturbance observer and a new partial tracking error constraint. *IEEE Transactions on Fuzzy Systems*.

[23] Ghanbari R., Ghorbani-Moghadam K., Mahdavi-Amiri N. (2019). A variable neighborhood search algorithm for solving fuzzy number linear programming problems using modified kerre’s method. *IEEE Transactions on Fuzzy Systems*.

[24] Shi Z., Sun X., Cai Y., Yang Z. (2020). Robust design optimization of a five-phase PM hub motor for fault-tolerant operation based on taguchi method. *IEEE Transactions on Energy Conversion*.

[25] Yin F., Zhao Y. (2021). Optimizing vehicle routing via Stackelberg game framework and distributionally robust equilibrium optimization method. *Information Sciences*.

[26] Liu X. (2021). Feature recognition of English based on deep belief neural network and big data analysis. *Computational Intelligence and Neuroscience*.

